# Taxonomic Precision of Different Hypervariable Regions of 16S rRNA Gene and Annotation Methods for Functional Bacterial Groups in Biological Wastewater Treatment

**DOI:** 10.1371/journal.pone.0076185

**Published:** 2013-10-16

**Authors:** Feng Guo, Feng Ju, Lin Cai, Tong Zhang

**Affiliations:** Environmental Biotechnology Laboratory, The University of Hong Kong, Hong Kong SAR, China; Rochester Institute of Technology, United States of America

## Abstract

High throughput sequencing of 16S rRNA gene leads us into a deeper understanding on bacterial diversity for complex environmental samples, but introduces blurring due to the relatively low taxonomic capability of short read. For wastewater treatment plant, only those functional bacterial genera categorized as nutrient remediators, bulk/foaming species, and potential pathogens are significant to biological wastewater treatment and environmental impacts. Precise taxonomic assignment of these bacteria at least at genus level is important for microbial ecological research and routine wastewater treatment monitoring. Therefore, the focus of this study was to evaluate the taxonomic precisions of different ribosomal RNA (rRNA) gene hypervariable regions generated from a mix activated sludge sample. In addition, three commonly used classification methods including RDP Classifier, BLAST-based best-hit annotation, and the lowest common ancestor annotation by MEGAN were evaluated by comparing their consistency. Under an unsupervised way, analysis of consistency among different classification methods suggests there are no hypervariable regions with good taxonomic coverage for all genera. Taxonomic assignment based on certain regions of the 16S rRNA genes, e.g. the V1&V2 regions – provide fairly consistent taxonomic assignment for a relatively wide range of genera. Hence, it is recommended to use these regions for studying functional groups in activated sludge. Moreover, the inconsistency among methods also demonstrated that a specific method might not be suitable for identification of some bacterial genera using certain 16S rRNA gene regions. As a general rule, drawing conclusions based only on one sequencing region and one classification method should be avoided due to the potential false negative results.

## Introduction

The activated sludge process is a key process in wastewater treatment that removes organic pollutants and nutrients (N and P) by the action of certain bacteria. Such bacteria include heterotrophic organic-utilizing bacteria, ammonia oxidizing bacteria (AOB), nitrite oxidizing bacteria (NOB), denitrifiers, polyphosphate accumulating organisms (PAOs), and others [1]. Many of these bacteria perform very specific functions (like AOB and NOB), and their activity is positively correlated with treating efficiencies [2], [3], [4]. These organisms are not only crucial in wastewater treatment plants (WWTPs), but also playing key roles in biogeochemistry [5]. This group of bacteria will hereafter be referred to as functional remediators. However, some other bacteria may cause operational problems to WWTPs. These include foaming and bulking bacteria, which may hamper solid-liquid separation [6], [7], [8]. This group of bacteria will hereafter be referred to as the operational group. In addition, the occurrence and abundance of bacterial pathogens in activated sludge is also a concern, since it may be highly correlated with occurrence of pathogens in effluent discharged into receiving water [9], [10], [11]. Generally, the performance and environmental impacts of WWTPs are significantly affected by the above three functional groups Identifying them at the sufficient taxonomic level (e.g., genus level) is significantly important for investigations on either microbial ecology of both full-scale plants and lab-scale reactors or linking microbial community with sewage treatment.

During the last two decades, molecular methods have enhanced analysis of bacterial communities and their functions in activated sludge. Various detection methods of bacterial identification that use the 16S rRNA gene have been developed. These include fluorescence *in situ* hybridization (FISH) [12], denaturing gradient gel electrophoresis (DGGE) [13], terminal-restriction fragment length polymorphism (T-RFLP) [14], clone library construction [15], quantitative PCR (qPCR) [16], and microarray [17]. Such methods have aided in revealing invaluable knowledge of the microbial ecology of activated sludge. However, these methods usually target certain bacterial groups or profile communities without much detail or resolution. For complex bacterial communities like activated sludge, traditional molecular methods could not provide sufficient resolution to adequately characterize the bacterial community function. For example, the abundances of AOB and NOB usually represent less than 1% of the total bacterial population in activated sludge from full-scale WWTPs, but they are extremely important for nitrogen removal [18].

Currently, high throughput sequencing techniques have proved their superiority for profiling complex microbial communities [19], [20]. For engineering applications, the profiling of the functional groups is more useful than enumerating the entire bacterial community. Monitoring those functional bacterial groups with high throughput sequencing may be served as routine analysis of WWTPs components, at reduced cost and greater efficiency. However, there are a number of limitations in analyzing 16S rRNA by current high throughput sequencing methods such as 454 pyrosequencing and Illumina paired-end sequencing. There are nine hypervariable regions separated by the conserved regions across the full length 16S rRNA gene. The sequence length, i.e. ∼450 bp for 454 pyrosequencing and ∼200 bp for Illumina paired-end sequencing could only cover two or three consecutive hypervariable regions and several single regions of 16S rRNA gene, respectively [21], [22]. On one hand, a number of reports had revealed that the selected primer sets targeting partial 16S rRNA gene suffered from biases among taxa due to their coverage [23], [24]. On the other hand, the short read length undoubtedly increases the uncertainty in taxonomic assignment [25]. Unfortunately, most specific functional microorganisms are required to be classified into genus level at least and the results at higher taxonomic levels are much less meaningful for the correlation of bacterial community and functions. Consequently it would be valuable to study which regions may be more suitable for identification of functional bacterial groups as precise as possible. So far, a number of studies had evaluated the coverage the primers for different hypervariable regions of 16S rRNA gene. Few of them analyzed the taxonomic precision for various regions [26]. Moreover, *in silico* datasets usually derived from the full length sequences are used for evaluation. These datasets may not exactly reflect the experimental results. Also, for specific and significant bacterial groups, such as the functional groups focused by the present study, there is no systematic evaluation to fully check the taxonomic precision of their partial 16S rRNA gene sequences.

In addition, the taxonomic annotation of sequences was realized by specific classification tools. The three classification methods commonly used in 16S rRNA gene-based prokaryotic taxonomic analysis are Ribosomal Database Project (RDP) Classifier based on the Bayesian algorithm, BLAST-based best-hit (BH) output that only considers the similarity between query and reference, and MEtaGenome ANalyzer (MEGAN) using BLAST-based lowest common ancestor (LCA) algorithm, which cares both the similarity between query and reference and the inconsistency of results with close or the same similarity [27]. In some cases, these three tools may give inconsistent results for some taxa, especially using short reads [18], [28], which will undoubtedly mislead some judgments. Thus they should be assessed for the identification of functional groups in activated sludge.

With an overall microbial community profiling, we recently reported that pyrosequencing of V3V4 amplicon of 16S rRNA gene obtained the highest bacterial diversity of a mixed AS sample [29]. However, in this study, the same pyrosequencing data were revisited to answer the following two questions: 1) what is the optimal region for precise taxonomy of those functional genera in AS samples? 2) How the biases are if using different classification methods? Since the real bacterial community in the sample is totally unknown, we chose to unsupervisedly compare the classification consistency among different classification methods to evaluate the taxonomic accuracy of different hypervariable regions. It is well-understood that the consistently assigned results are more likely correct. The results will be helpful not only for the methodology of studying on microbial ecology in activated sludge and other complex environmental samples by high throughput sequencing, but also for routine monitoring of the functional bacterial groups in activated sludge. More importantly, the results will be informative to alert known biases when using pyrosequencing to profile the bacterial community in activated sludge or other systems by different sequencing region or classification methods.

## Materials and Methods

### List of functional groups

The assemblage of species in each functional group was selected based on previous reports 1,10, as shown in Table 1. Bacteria in the remediator group include typical and well-known genera of AOB, NOB, denitrifiers, PAOs, glycogen accumulating organisms (GAOs) and hydrolysers. As to the bulking and foaming bacteria group, only the formally named genera were recruited. Members of the pathogenic group, were classified as ‘potential pathogens’, since in many cases the 16S rRNA gene can only be used for the identification to the genus level, while the pathogens should be identified to the species at least. In total, the list contains 73 genera, including three multifunctional genera, *Rhodococcus*, *Mycobacterium* and *Tetrasphaera* that were assigned to more than one group. Moreover, there are three candidate genera, ‘*Accumulibacter*’ ‘*Competibacter*’ and ‘*Microthrix*’, which could not be recognized by the RDP Classifier or the LCA method due to limitation of current databases. They can only be assigned by the BH method.

**Table 1:** List of functional groups in activated sludge.

Functional group	Subgroup	Genus^a^	Reference sequence number in database^b^
**Nutrient remediators**	AOB	*Nitrosomonas*	48/78(61.5%)
		*Nitrosospira*	68/68(100%)
		*Nitrosococcus*	19/19(100%)
	NOB	*Nitrobacter*	73/80(91.3%)
		*Nitrospira*	21/21(100%)
	Denitrifier	*Acidovorax*	208/233(89.3%)
		*Arcobacter*	92/92(100%)
		*Azoarcus*	57/64(89.0%)
		*Comamonas*	221/244(90.6%)
		*Curvibacter*	12/14(85.7%)
		*Dechloromonas*	31/34(91.2%)
		*Hyphomicrobium*	61/65(93.8%)
		*Meganema*	2/2(100%)
		*Methylobacillus*	10/13(76.2%)
		*Methylophilus*	26/29(89.7%)
		*Paracoccus*	308/318(96.9%)
		*Rhodobacter*	99/137(72.3%)
		*Rhodococcus*	783/800(97.9%)
		*Thauera*	61/61(100%)
		*Zoogloea*	31/36(86.1%)
	PAO	*‘Accumulibacter’*	0/12(0%)
		*Tetrasphaera*	21/21(100%)
	GAO	*‘Competibacter’*	0/1(0%)
		*Defluviicoccus*	2/2(100%)
	Hydrolyser	*Saprospira*	4/11(36.4%)
		*Lewinella*	19/19(100%)
**Bulking and foaming group**	Bulking bacteria	*‘Microthrix’*	18/18(0%)
		*Acinetobacter*	1,677/1,690(99.2%)
		*Aquaspirillum*	4/11(36.4%)
		*Beggiatoa*	10/26(38.5%)
		*Caldilinea*	2/2(100%)
		*Flexibacter*	22/74(29.7%)
		*Gordonia*	223/225(99.1%)
		*Haliscomenobacter*	7/8(87.5%)
		*Leucothrix*	4/5(80%)
		*Moraxella*	84/123(68.3%)
		*Sphaerotilus*	16/16(100%)
		*Thiothrix*	70/72(97.2%)
		*Trichococcus*	40/40(100%)
	Foaming bacteria	*Isosphaera*	2/12(16.7%)
		*Mycobacterium*	1,063/1,068(99.5%)
		*Nocardia*	768/777(98.8%)
		*Rhodococcus*	783/800(97.9%)
		*Skermania*	12/12(100%)
		*Tetrasphaera*	21/21(100%)
**Potential pathogen**		*Aeromonas*	768/770(99.7%)
		*Bordetella*	186/192(96.9%)
		*Borrelia*	322/322(100%)
		*Brucella*	187/201(93.0%)
		*Campylobacter*	450/452(99.6%)
		*Chlamydia*	72/78(92.3%)
		*Chlamydophila*	30/30(100%)
		*Clostridium*	1,586/1,694(93.6%)
		*Corynebacterium*	614/624(98.4%)
		*Escherichia*	710/731(97.1%)
		*Enterococcus*	843/856(98.5%)
		*Francisella*	100/101(99.0%)
		*Haemophilus*	882/964(91.5%)
		*Helicobacter*	415/418(99.3%)
		*Klebsiella*	517/806(64.1%)
		*Legionella*	193/206(93.7%)
		*Leptospira*	315/316(99.7%)
		*Listeria*	190/190(100%)
		*Mycobacterium*	1,063/1,068(99.5%)
		*Mycoplasma*	356/533(66.8%)
		*Neisseria*	1,229/1,235(99.5%)
		*Pseudomonas*	5,819/6,057(96.1%)
		*Rickettsia*	164/164(100%)
		*Salmonella*	339/365(92.9%)
		*Serratia*	629/652(96.5%)
		*Shigella*	288/288(100%)
		*Staphylococcus*	1,207/1,214(99.4%)
		*Streptococcus*	2,289/2,311(99.0%)
		*Treponema*	1,074/1,089(98.6%)
		*Vibrio*	1,533/1,705(89.9%)
		*Yersinia*	311/315(98.7%)
**Total**			29,930/31,522(94.9%)

a. Genus names with single quote marks were candidate genera that could not be classified by RDP classifier. They are kept in the final database.

b. The first number indicates the sequences that could be identically classified in RDP at genus level (except for the candidate genera) and thus are kept in the final database. The second is the number of total downloaded sequences from Greengenes database. Percentage showed the portion of identically classified sequences.

### Modified Greengenes database using validated sequences of the functional groups

Nearly full length 16S rDNA sequences (>1,250 bp and 1,444 bp in average) of all the above 73 genera were manually downloaded from Greengenes online database [30]. After that, the identity assigned by RDP classifier (at 80% confidence threshold cutoff) was compared with the original Greengene assignments of all these sequences. If the RDP results were inconsistent with the actual identity for one sequence, the set was removed from the downloaded dataset (about 5.1% sequences were eliminated). The remaining sequences were added to the pre-cleaned Greengenes database (released at May 9, 2011, totally 385,791 reference sequences with mean length of 1,405 bp), from which the 73 genera listed in Table 1 had been removed. This was done to produce a more comprehensive and updated database to use in the BLAST search in this study. After removing redundancies, the modified Greengenes database contained 415,721 sequences, with 29,930 sequences belonging to the selected functional groups (Table 1).

### Activated sludge sample preparation, pyrosequencing and data processing

FastDNA® SPIN Kit for Soil (MP Biomedicals, France) was used to extract total DNA from the eleven activated sludge samples [29], according to manufacturer's instructions. We confirm that: i) the sampling sites were not privately-owned or protected in any way; and ii) the field studies did not involve endangered or protected species. This kit has been found to be the most efficient method for extracting DNA from activated sludge [31]. Equal masses of DNA from different samples were mixed for the following PCR amplification. The primers used in PCR and their coverage for domain *Bacteria* determined by RDP ‘Probe Match’ were listed in Table S1 in file S1. The details for barcoded-PCR and pyrosequencing can be found in reference [29]. We sequenced the same amplicon from both directions. For instance, two types of pyrotags, i.e. V12 (sequencing from V1 to V2) and V21 (sequencing from V2 to V1) were obtained for V1&V2 regions and distinguished by the differently barcoded forward and reverse primers. The two sub-datasets for the same amplicon hypervariable regions were analyzed as duplications below.

After the raw reads were obtained, they were quality-checked to remove all reads with any ambiguous bases. Then the barcodes and primers were trimmed from the reads in RDP Pyrosequencing Pipeline. The average sequencing length of reads (for those regions over 400 bp) was approximately 400∼430 bp, most reads could fully cover the V1&V2 (∼350 bp) and V5&V6 (∼300 bp) amplicons, but reads only partially covered the V3&V4 (440∼470 bp) and V7&V8&V9 (∼420 bp) amplicons. To avoid bias caused by trimming with both primers, we trimmed the reads of V3&V4 and V7&V8&V9 only with the single primer (at the beginning of sequencing). Two mismatches for each primer were allowed during trimming and only trimmed tags over certain length (V5&V6, >200 bp; V1&V2, >250 bp; V3&V4 and V7&V8&V9, >300 bp) were kept for further analysis. Then the tags were further cleaned by Denoise [32] and Chimera Check [33] using the software package of MOTHUR 1.22 [34]. After the eight sets of cleaned pyrotags were obtained, they were randomly normalized at the same depth of 32,000 each, except for V987 at 24,000. The raw. *sff* files for this study were uploaded to the Sequence Read Archive of NCBI (SRR790735).

### Evaluation of taxonomic precision of different sequencing regions and classification methods

To evaluate taxonomic accuracies of the different variable regions, we compared the consistency of results from three widely used 16S-rDNA-based taxonomic assignment methods: 1) online RDP Classifier [25], 2) BH output, and 3) MEGAN -LCA output after BLAST against the modified Greengenes database. We assumed that higher consistency among different methods indicated higher precision of taxonomic assignment for that variable region. Moreover, the more consistently classified tags suggest the higher efficiency derived from both taxonomic capability and PCR recovery (i.e., primer coverage).

In most cases the confidence threshold for RDP classifier was set at 80% if not mentioned. For the BLAST against the modified Greengenes database, the expect-value (e-value) was set at 10^−100^ and the top 50 hits were kept in the output file. The e-value was approximately equal to 85% similarity for a 300 bp alignment length. To filter the low similarity hits, the results were edited by eliminating all hits with less than 95% (for LCA annotation) or 97% (for BH output) similarity. Because the IDs of the DNA sequences from Greengenes in the BLAST output file are Greengenes No., not names of taxa, a script was written in Python to transform the Greengenes IDs into the lowest taxon recorded in the modified Greengenes database. For the BH method, a Python script was used to extract the first output line (with the highest bit score) for each type of pyrotag, and the abundance of all functional genera was counted by another script. The LCA annotation by MEGAN (Version 4.70.4) was performed using the default parameters except that ‘Min Support’ (the hit number cutoff for output) was set at 1 instead of 5. The files containing read names and their corresponding taxonomic names were output at genus level for all the three methods. For each genus and variable region, the number of pyrotags was concordantly classified by all three methods, by two of the three methods, or by only one of the methods were quantified using a Python script.

## Results

### Database construction

Table 1 showed the functional groups (including remediators, operational and pathogenic groups) of bacteria in activated sludge. In total, 31,522 nearly full-length 16S rDNA sequences belonging to the 73 genera were downloaded from Greengenes database, therein 29,930 (94.9%) of them could be correctly identified by RDP Classifier at confidence threshold of 80%. For most genera, the eliminated sequences represented only a small portion of the total (<10%). However, For *Nitrosomonas*, *Beggiatoa*, *Klebsiella* and *Mycoplasma*, more than 20% were eliminated. For *Saprospira*, *Flexibacter*, *Aquaspirillum* and *Isosphaera*, over half of the reference sequences were eliminated.

### Evaluation of hypervariable regions

**Figure 1:**
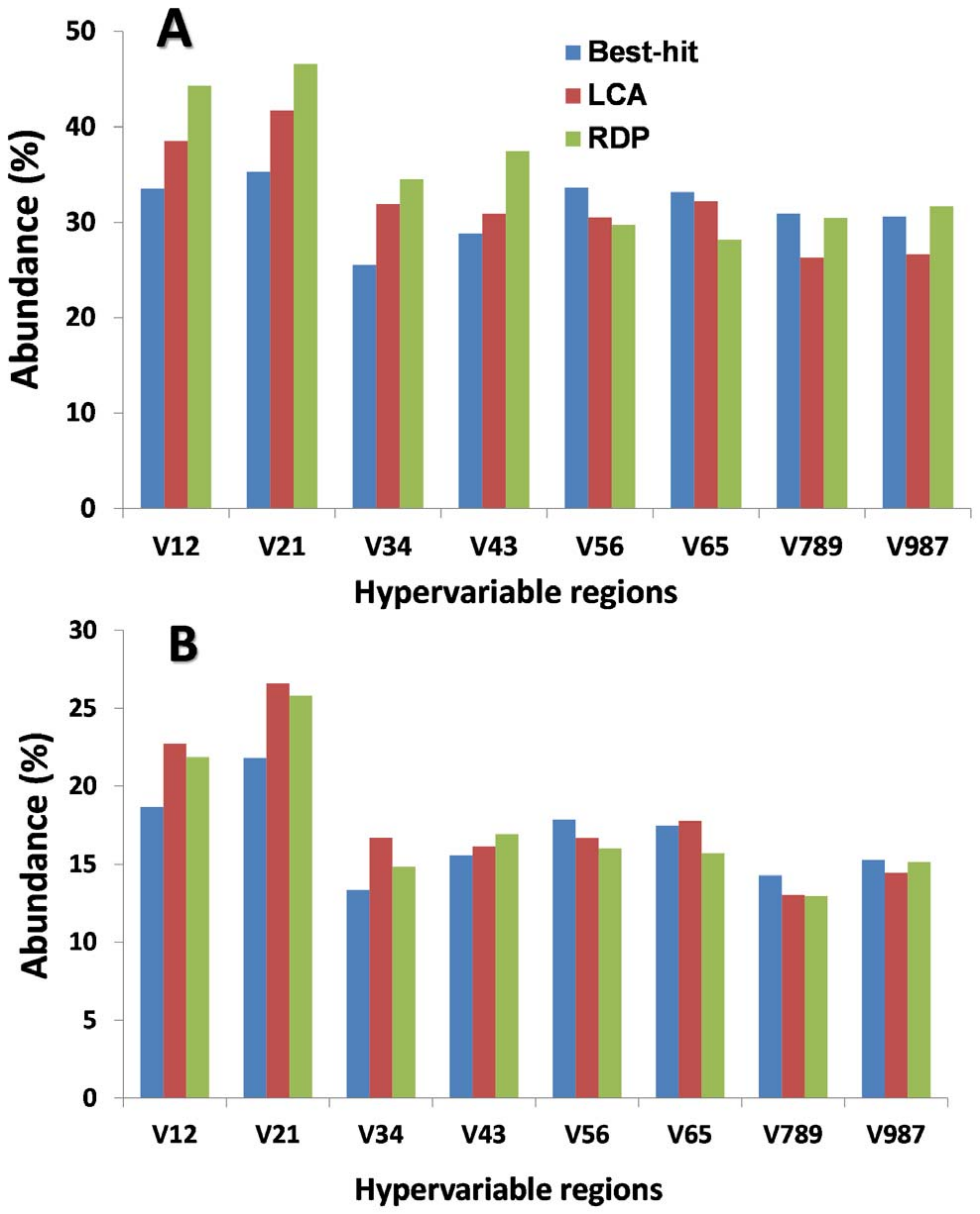
Percentages of pyrotags that could be assigned at genus level (A) and concerned functional genera (B) by three annotation methods of RDP Classifier, Best-hit and LCA.

The percentages of sequences that could be assigned into any certain genus and all functional groups are shown in Fig. 1A and B, respectively. About 28.2%∼46.6% reads could be assigned into a genus at 80% confidence threshold in RDP Classifier, while approximately 25.5%∼35.3% and 26.3%∼41.7% reads were assigned to a genus for BH and LCA methods, respectively (Fig. 1A). For functional groups, as shown in Fig. 1B, bacteria belonging to the functional groups accounted for 12.6%∼25.8% of total bacteria based on the RDP results. Also approximately 42.5%∼55.7% of the sequence tags were assigned to genus level. These results suggest that roughly half of the genera present did not belong to the listed functional groups, and their roles in activated sludge may need further exploration.

On average, analysis by all three methods indicated that genus-level assignment was the most effective when using the V12 and V21 sequences. This was true when including all species and when only using bacteria belonging to the functional groups. The V34 and V789 regions had the least reads assigned by BH and LCA methods, respectively. Interestingly, discrepancies between RDP and BLAST-based methods for functional groups were not as large as those for the total assemblage. This suggested that the three methods were more consistent when focusing on the functional groups than when including all species. It may be due to the fact that bacteria in the functional groups are usually well-characterized compared with most other bacterial genera, which makes them more likely to be classified correctly.

**Figure 2:**
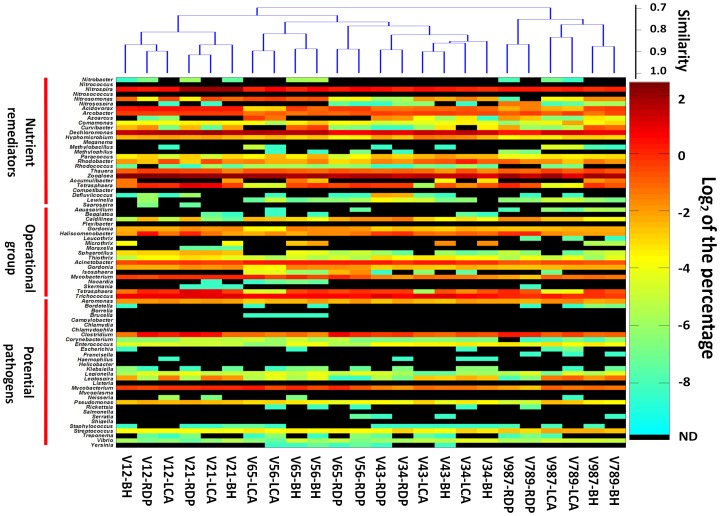
Percentages of pyrotags identified as concerned genera for various multi-variable-regions by three methods. The cluster among columns was based on the mean Bray-Curtis distances calculated by the percentages of each genus to total pyrotags. The outer, meddle and inner rings are corresponding to remediators, operational group and potential pathogens.

The percentages of members of the functional groups are displayed using a heat map in Fig. 2 according to 16S rRNA gene region and classification method used. Five genera, such as *Nitrococcus*, ‘*Competibacter*’, *Campylobacter*, *Chlamydophila* and *Helicobacter* were not detected for all regions and classification methods used (detection limit at 0.003%). Two candidate genera, ‘*Microthrix*’ and ‘*Accumulibacter*’ were only detected by the BH method because they were not included in the RDP taxonomic system and MEGAN-LCA annotation. There were 45 to 58 genera that belonged to the functional groups, depending on the 16S rRNA gene region and classification method used. Four genera with >1% average abundance were *Zoogloea* (3.53%), *Dechloromonas* (2.47%), *Nitrospira* (1.53%) and *Trichococcus* (1.52%). Because the mixed activated sludge samples were collected from eleven municipal WWTPs located in several countries, these highly abundant genera represented the common key groups in municipal activated sludge. For example, *Zoogloea*, one of the most common genera, is not only a denitrifier, but also a specific floc-forming bacterium, and may be important in the organization of activated sludge [35].

The results from cluster analysis of the classification results using different regions, sequencing direction, and annotation methods, is also shown in Fig. 2. These results indicate that sequences were clustered first according to regions. However, except for the RDP results, V56 and V65 clustered with the V34 region. Moreover, there was usually less than 80%∼90% similarity among results determined by different classification methods of the same sequencing regions, indicating the classification methods could cause noticeable biases as well as the sequencing regions.

**Figure 3:**
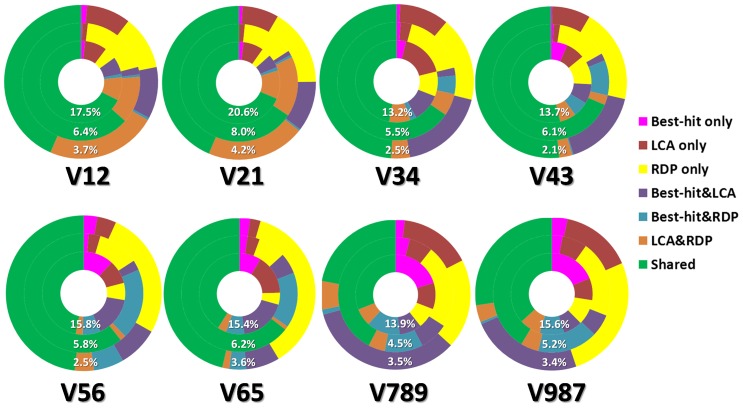
Agreement of pyrotags assigned as functional bacteria among RDP classifier, BH and LCA methods. The inner, middle and outer rings are corresponded to the remediators, operational groups and potential pathogens, respectively. The percentages in the pies are the ratios of pyrotags assigned by all the three methods to total tags. Higher shared tag portions and percentage of assigned pyrotags suggested higher efficiencies of the variable regions.

The classification consistencies using different multi-variable regions were evaluated by comparing results from the three classification methods and were summarized in Fig. 3. Results were classified according to 3 categories: 1) Those consistently assigned to the same genus by all three methods 2) those assigned to the same genus by two different methods and 3) those assigned to a genus by only one classification method. Obviously, sequence tags that were consistently classified tags by all three methods or by two methods are more likely correct than those exclusively assigned. Using this criterion, the V1&V2 (average of V12 and V21) and V3&V4 region is good for classifying all the functional group. And V1&V2 is slightly better than V3&V4 due to its high percentage of consistently assigned tags for remediators. On the other hand, the V7&V8&V9 region is not recommended for any of the functional groups, due to a large proportion of inconsistently-assigned tags.

**Figure 4:**
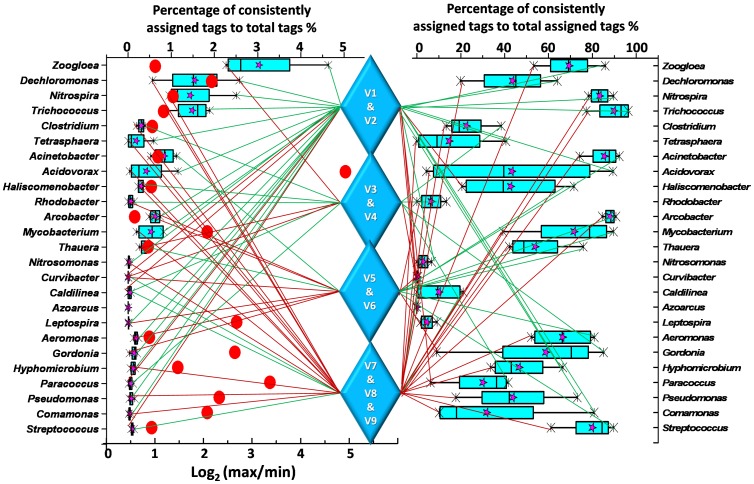
Comparison of consistently assigned tags by 3 methods among different sequencing regions for the moderately abundant genera. In the left box chart, abundance is the percentage of consistently assigned tags for 4 regions (V1&V2, V3&V4, V5&V6 and V7&V8&V9, abundance of V1&V2 is the mean value of V12 and V21). Only 25 genera which were over 0.1% abundance as determined by at least one method were listed. *Azoarcus* had no consistently assigned tag although its abundance is high as determined by LCA annotation. Red dots showed the logarithmetics of the ratios of maximum identified tags to minimum ones among different regions. No dots for *Nitrosonomas*, *Caldilinea*, *Curvibacter* and *Azoarcus* because they get at least one 0-hit region. Percentages of 3-method consistently assigned tags to total assigned tags for each region were compared in the right box chart. Shown are the minimum, 25% quantile, median, 75% quantile and maximum. Stars indicated the average value. For each genus, colored lines link to the most abundant/consistently assigned region (green) and the least abundant/consistently assigned region (red). More links of green lines indicated the priority of the regions, such as V1&V2, while more red ones suggested the poor performance of the region (like V7&V8&V9) for these abundant genera.

Other than the overall presentation of Fig. 3, Fig. 4 summarized the most and the least efficient regions for moderately abundant genera (over 0.1% abundance as determined by at least one method), supporting by the abundance of consistently assigned tags (see the left box chart). It is obvious that no region could have a good coverage for all 25 genera since every region have the several lowest-classified genera. For different genera, abundances of consistently assigned tags fluctuated from 1.5 to 30.4 folds among different regions, if not considering the undetected genera in certain regions (*Nitrosonomas*, *Caldilinea*, *Curvibacter* and *Azoarcus*). It was suggested that quantification of those functional genera might be seriously biased with a bad choice of hypervariable regions. The variation of quantification results should be derived from either primer coverage (PCR efficiency) or taxonomic precision of different segments. The abundance of consistently assigned tags was affected by both primer coverage and taxonomic precision of the selected region. However, the consistencies among methods only potentially reflect the taxonomic precision of specific hypervariable regions. In general, agreed with Fig. 3, V1&V2 showed relatively high efficiency, regarding to both the abundance of consistently assigned tags (left box chart) and the consistency among methods (right box chart). Many genera (10 genera), especially for most of the high-ranked genera (six of top ten genera), got the best consistent assignment while only 4 (*Caldilinea*, *Curvibacter*, *Paracoccus* and *Haliscomenobacter*) got the poorest consistencies for V1&V2. On the contrary, it could be drawn that V7&V8&V9 is not recommended for profiling functional bacteria in activated sludge due to too many genera get the fewest consistently assigned tags (13 genera) in this region and the poorest assignment consistency (16 genera) for the 25 genera.

Further, we estimated how uncertain the inconsistent assigned results were, i.e. whether the sequences could be correctly assigned at higher taxonomic levels (e.g. family and order). The most inconsistently assigned genus (see Fig. 4), *Acidovorax* were only analyzed here. As illustrated in Fig. S1 in file S1, the tags that could only be solely assigned by LCA or BH were checked by RDP Classifier at the confidence threshold of 80%. In most cases, the assignments were correct at order levels except for the V3&V4 sequences that were solely assigned as *Acidovorax* by LCA. The V789 amplicons were often mis-annotated at family level. The V1&V2 and V5&V6 regions exhibited well accuracy at family level, suggesting the relatively higher taxonomic accuracy for this family on the other side.

### Evaluation of different classification methods

**Figure 5:**
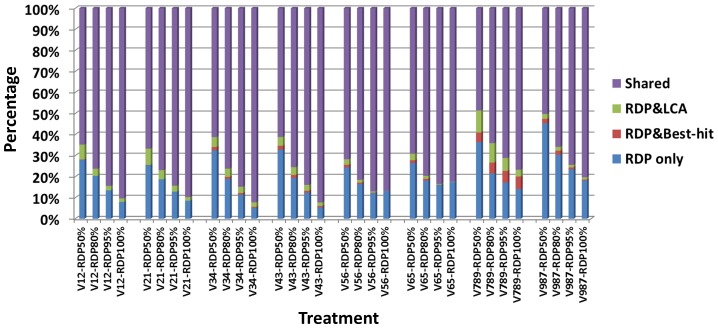
The consistencies between the RDP-classifier and other two methods under different confidence thresholds for different multi-variable regions. Raw results from RDP classifier were filtered with different confidence thresholds by a Python script, and then compared with the other two methods through another script. The percentages of consistently classified tags were increased with more strict thresholds, while the solely classified tags showed the opposite trend.

Firstly, since the tags solely assigned by RDP Classifier with 80% confidence threshold usually account for large portions of total assigned tags for all regions (Fig. 3), it is worth to figure out whether this method is precise enough under various confidence thresholds. We tested 50%, 80%, 95% and 100% thresholds and found that the consistency between RDP and other two methods was increased with the strictness (Fig. 5). Generally, about 30%∼50% classified tags were filtered out if increasing the confidence threshold from 50% to 100%. Among all the tags assigned as concerned groups, 23.9%∼45.2% of them were solely assigned by RDP at 50% confidence, while the ratios decreased to 5.3%∼18.1% at 100% confidence. The consistencies at 100% confidence threshold are relatively high in V3&V4 and V1&V2. The region of V7&V8&V9 showed the worst performance because there was about 20% reads assigned to the functional groups could not be recognized by BH and LCA.

Even more importantly, this result also suggested that the current version RDP Classifier may have some biases because many tags that were identified very surely (at 100% confidence) could not be agreed by BLASTn-based methods, which exhibit the real phylogenetic distances between sequences. Although the overall error rate for RDP Classifier is not high, it may introduce significant biases for some important groups, such as *Nitrosomonas* [18]. Under this consideration, a further investigation on the detected moderately abundant genera (>0.1% abundance at least in one of four multi-variable regions) was conducted. Here, two indexes, one for potential underestimation and the other for potential overestimation, were proposed. The former is the percentage of tags that were exclusively classified by BH and/or LCA to total classified tags and the latter is percentage of tags that were exclusively classified by RDP Classifier to total tags. Larger indexes (typically over 50%) suggested the higher potential biases. As listed in Table 2, there were some genera that could be underestimated or overestimated for all multi-variable regions. *Caldilinea* were always potentially over-counted, while *Nitrosomonas*, *Rhodobacter*, *Curvibacter* and *Leptospira* tended to be seriously underestimated no matter which multi-variable region was selected. It agreed with our previous report about mistakenly assigned the AOB genus *Nitrosomonas* by RDP [18]. Table 1 also showed *Nitrosomonas* and *Rhodobacter* got many inconsistent taxonomic assignments between Greengenes database and RDP Classifier even using full length of 16S rRNA gene. Further, we found that at stricter confidence thresholds the potential overestimation decreases dramatically, but the situation of underestimation becomes reasonably more serious, and *vice versa* for looser confidence threshold.

**Table 2:** Evaluation of taxonomic precision of RDP Classifier for the moderately abundant genera by referring to Best-Hit and the lowest common ancestor methods.

Genus	Abundance^a^ %	Index of potential overestimation^b^ %								Index of potential underestimation^c^ %							
		V12	V21	V34	V43	V56	V65	V789	V987	V12	V21	V34	V43	V56	V65	V789	V987
*Zoogloea*	4.28	2.9	3.9	9.5	8.2	1.7	1.6	3.9	3.3	5.7	6.1	14.8	7.0	20.0	28.6	31.0	19.9
*Dechloromonas*	3.41	1.7	1.5	2.1	4.0	0.3	0.3	2.0	1.5	20.5	21.7	43.2	33.5	**52.0**	**59.1**	**56.4**	**60.0**
*Nitrospira*	1.68	2.6	1.1	8.8	8.3	8.6	10.9	3.3	7.7	10.3	3.6	4.6	1.7	4.4	5.8	2.8	3.5
*Trichococcus*	1.62	2.8	2.2	2.8	1.8	0.4	1.7	8.7	2.2	0.2	0.0	4.0	1.6	0.9	3.5	9.6	10.5
*Clostridium*	1.44	22.9	30.5	37.4	42.7	**62.2**	**72.1**	43.6	**58.7**	9.0	7.7	22.2	16.4	6.7	4.6	31.9	23.5
*Tetrasphaera*	1.04	6.0	6.6	22.7	28.2	2.3	2.1	14.9	20.5	0.0	0.5	9.5	6.6	3.0	4.5	21.9	9.6
*Acinetobacter*	0.96	2.0	4.3	1.0	4.7	0.0	0.2	0.0	0.0	2.4	1.4	4.5	2.3	5.9	9.2	23.1	21.4
*Acidovorax*	0.94	2.7	1.5	1.0	0.0	**53.2**	24.9	6.0	3.0	6.4	6.2	36.8	22.8	14.4	24.6	**63.7**	**75.7**
*Haliscomenobacter*	0.83	**70.5**	**63.4**	42.7	39.6	23.1	32.7	**71.3**	**67.6**	0.0	0.0	0.0	0.4	0.0	0.0	0.3	0.0
*Rhodobacter*	0.81	0.7	0.0	5.6	9.6	22.7	19.7	29.3	22.8	**82.5**	**83.2**	**79.2**	**56.3**	**76.8**	**80.3**	40.8	36.6
*Arcobacter*	0.71	6.4	3.1	2.5	5.1	6.8	10.5	1.6	7.0	5.3	3.1	4.5	5.1	0.5	0.7	2.9	6.2
*Mycobacterium*	0.70	0.0	0.6	0.0	0.0	0.0	0.3	0.0	0.0	16.9	12.7	26.2	23.1	9.3	11.2	**63.5**	**58.0**
*Thauera*	0.70	1.5	4.5	7.7	9.4	0.0	2.8	5.7	2.4	6.7	5.0	16.8	10.0	6.9	10.6	40.9	36.1
*Nitrosomonas*	0.55	0.0	0.9	0.0	5.1	4.6	1.2	0.0	0.0	**89.9**	**85.9**	**93.9**	**93.9**	**89.4**	**92.2**	**100.0**	**98.9**
*Curvibacter*	0.47	61.1	39.2	3.2	2.6	0.8	2.4	2.5	19.9	32.9	**51.2**	**96.8**	**97.4**	**98.9**	**97.6**	**96.9**	**71.7**
*Caldilinea*	0.32	**85.9**	**93.8**	**70.1**	**61.1**	**62.3**	48.3	**51.0**	**52.6**	14.1	6.3	3.7	2.8	23.9	45.8	4.9	3.9
*Azoarcus*	0.31	0.0	N.D.	**69.4**	**77.5**	0.0	0.0	**70.6**	**65.3**	**50.0**	N.D.	24.1	18.6	**100.0**	**100.0**	26.5	13.3
*Leptospira*	0.28	0.0	0.0	3.0	7.3	0.0	0.0	0.0	0.0	**94.8**	**94.7**	**95.5**	**85.5**	**94.1**	**87.5**	**97.8**	**98.9**
*Aeromonas*	0.28	0.0	4.3	0.0	1.6	0.0	0.0	0.0	1.4	11.2	19.6	15.6	17.7	18.2	7.9	41.7	23.3
*Gordonia*	0.27	2.5	6.5	0.0	0.0	0.0	0.0	0.0	0.0	19.0	5.6	7.7	14.0	21.7	29.5	36.0	22.4
*Hyphomicrobium*	0.25	3.8	3.6	3.8	1.3	0.0	0.0	12.5	7.0	20.5	18.1	**54.8**	49.7	**50.8**	**51.1**	39.3	**53.5**
*Paracoccus*	0.19	28.3	25.8	2.8	12.0	0.0	2.9	0.0	0.0	13.2	27.4	48.6	**50.0**	**61.5**	**68.6**	**54.5**	**60.0**
*Pseudomonas*	0.16	9.1	10.3	0.0	0.0	0.0	0.0	0.0	0.0	6.1	8.8	45.9	40.9	42.9	57.1	**76.9**	**81.4**
*Comamonas*	0.14	14.3	0.0	8.3	3.4	21.2	14.3	2.3	0.0	10.7	3.3	**58.3**	**62.1**	**57.6**	46.9	**85.1**	**90.0**
*Streptococcus*	0.13	10.7	16.0	0.0	0.0	0.0	0.0	0.0	0.0	3.6	0.0	6.8	7.1	11.1	9.4	40.0	34.5

a Twenty-five genera accounted over 0.1% average abundance determined by all three methods were listed.

b Index of potential overestimation is the percentage of tags that were exclusively classified by RDP classifier to total classified tags for certain genus. The confidence threshold for RDP classifier is 80%. The boldface number stressed those seriously underestimated or overestimated regions with index over 50%.

c Index of potential underestimation is the percentage of tags that were exclusively classified by BH and/or LCA to total classified tags.

Further, we also compared the divergence between BH and LCA for different multi-variable regions. The results were presented in Table S2 in file S1. The two BLAST-based methods also exhibited divergences. The pyrotags that could be assigned by BH but not by LCA should be resulted from the share of similar partial sequence of references among different taxa. Interestingly, in most cases this type of inconsistency existed in certain regions. For instance, V1&V2 exhibited high capability in classification of *Tetrasphaera*, while other regions were mostly LCA-negative. On the other hand, there are many tags only could be assigned by LCA. It indicated many tags are over 95% but less than 97% similarity to the references. Their phylogenetic position is still hard to be evaluated. They may be the uncharacterized species or genera closely related to the references.

## Discussion

For factors affecting on bacterial consortia profiling by high throughput sequencing, current methodological investigations mainly focused on DNA extraction [31] and primer coverage [29], [36]. The shortages from short read length are not often mentioned partially because the technique unprecedentedly refined the resolution of community profile and quantitative capability. However, in order to achieve the best taxonomic resolution for certain groups, the selection of sequencing regions and classification methods should be evaluated. Therefore, the real purpose for our study is to avoid the wrong or insufficient taxonomic assignments for those sequences that were truly generated from certain genera as possible and to get pre-evaluation of the potential biases for the concerned genera. Noticeably, these biases had been luckily amended in some studies and may be undermined in the others [18], [28].

Many studies had examined the classification capability of different variable region [23], [26], [37]. However, most of them analyzed the consistency between the artificially cut partial segments and their mother full-length sequences, which are not really generated by PCR. More importantly, all the previously reports targeted the overall classification without considering the weight of those significant functional groups. Recently, a report surveying 16S rRNA gene sequences from many environments showed that there was no perfect primer sets to cover all the examined sources [38]. The authors proposed that validation of primer sets (or regions) must be pre-evaluated for specific samples. However, besides matching of primers, obviously the different regions of 16S rRNA gene sequences and annotation methods have different validities for specific genera. Although our study only focused on the highly concerned functional genera in wastewater treatment systems, the results suggested that even for a specific sample, it is still hard, even impossible to get unbiased taxonomic information due to primer coverage, PCR amplification, taxonomic precision of the various sequencing regions and different annotation methods. To get pre-evaluated information for the highly concerned bacterial taxa rather than sample types is an alternative way to avoid significant biases.

For the selection of pyrosequencing region to analyze major functional bacterial community in activated sludge, based on both the abundance of consistently classified tags and the consistencies among methods, V1&V2 is the preferred region and V7&V8&V9 is the worst choice of 16S rRNA gene pyrosequencing. V1&V2 is very suitable in length for current pyrosequencing (around 350 bp amplicon). This primer set had been widely adopted in many previous studies among various sample types [39], [40]. Nevertheless, we also found that several functional genera would be underestimated or got the poorest taxonomic consistency in this region, such as *Caldilinea*, *Curvibacter*, *Hyphomicrobium* and *Streptococcus*.

The result is thought to be somewhat contradictive with our previous report that V3&V4 region is the most powerful for bacterial diversity profiling of activated sludge [29]. Thus, it was raised a concerning on whether there was a perfect selection of variable regions for pyrosequencing of 16S rRNA gene for different intentions, such as investigations on overall community or specific groups. Our results suggested that the selection would be never perfect even within very limited groups (genera). It also reminded us that the classification results may be ‘fake’ or ‘blurred’. To get pre-evaluation for the most important bacteria will be helpful for judgments.

Another interesting result is that the V1&V2 region has the most classified pyrotags at genus-level for overall community and functional groups. We found that the used primer set for this region is the only one without any degenerated base. Using degenerated base for primer will expand their coverage undoubtedly. However, whether it affects the quantification need further tests.

The precision and accuracy of classification methods are highly dependent on their databases. It should be noted that the RDP Classifier and LCA algorithm will be very precise if their databases are perfect since they are tree-based and supervised, respectively. However, the Best-Hit method is only dependent on the similarity unsupervisedly, which could hardly be consistently correct by using a single cutoff for all bacteria. RDP classifier is very powerful in dealing with large datasets due to its simple algorithm and the high precision for long sequences under high confidence threshold. Regarding to the speed, online RDP Classifier could get the classification of the 32,000 pyrotags within a few minutes, while several days is needed for BLAST-based methods (against Greengenes database, with a single CPU of 2.8 G Hz). However, the RDP might make wrong assignments for short reads [25]. In this study, we found that RDP Classifier overestimated and/or underestimated several genera in some regions or even all regions. It should be highly careful to avoid drawing conclusions from the negative results based on the absence of specific genera, which are investigated by amplifying the inefficient region or using biased annotation method. The false negative results were much more misleading since significantly false positivity is hardly possible for the complex community like activated sludge. Thus, the problem of underestimation or even missing of certain genera using RDP Classifier, which could only be realized and solved by applying other methods, is more serious than overestimation. Moreover, it was found that some genera were innately less distinguishable by RDP Classifier no matter what regions were selected, such as *Nitrosomonas* and *Leptospira* (see Table 2).

On the other hand, BLAST-based methods also generated potential errors [28], [41]. For taxonomic assignment of the short 16S rDNA segments, these bugs are mainly derived from highly similar reference sequences (at specific variable regions) that belong to different taxa, even at genus or higher levels. The LCA algorithm that based on the agreement of qualified ‘voters’ was invented to solve this confuse [27]. Moreover, the reliability of BLAST-based taxonomy elementarily relies on the accuracy and integrity of database and suitable parameter setup. For example, the top 10% score-cutoff (default setting) for the LCA methods in MEGAN may require to be evaluated for the annotation on short tags.

In summary, high throughput sequencing of the segments of 16S rRNA genes is like taking high-resolution images for microbial community. However, current short read length that only cover a couple of hypervariable regions may make some ‘pixels’ defective due to PCR bias and low taxonomic precision. For studies on some specific groups, it may introduce large and unexplainable biases although these “pixels” may be insignificant in the studies about overall microbial communities. Thus pre-evaluation is needed to select the suitable regions and annotation methods for the concerned specific groups. This study focusing on the highly concerned bacterial genera in the activated sludge demonstrated an example for the pre-evaluation on different regions and classification methods. The results showed that there was no perfect region for all concerning abundant functional genera, except for some regions with less bias, i.e. V1&V2, which could be the preferred region of functional groups in activated sludge. On the other hand, the results also demonstrated the divergences among different classification methods, which may cause misleading results. To avoid drawing wrong conclusion from negative results based on only one region or method, cross-checking using multiple regions and annotation methods should be proposed for taxonomic assignment of pyrosequences of short 16S rRNA gene amplicons.

## Supporting Information

File S1 **File containing Figure S1, Tables S1 and S2.** Figure S1. Accuracies at family and order levels for the tags that solely assigned as *Acidovorax* by LCA (A) or BH (B) of different regions. The tags were extracted and then checked with RDP Classifier at confidence thresholds of 80%. The RDP results of *Comamonadaceae* at family level and *Burkholderiales* at order level were cataloged as accurate assignments. The V12 amplicons had no tag that was solely assigned by BH.
(DOCX)
