# Supplementary material for: Taxonomic Precision of Different Hypervariable Regions of 16S rRNA Gene and Annotation Methods for Functional Bacterial Groups in Biological Wastewater Treatment
Source: PLoS One. 2013 Oct 16;8(10):e76185. doi: 10.1371/journal.pone.0076185 (PMC3797802; doi:10.1371/journal.pone.0076185)
Supplement: File S1 — File containing Figure S1, Tables S1 and S2. Figure S1. Accuracies at family and order levels for the tags that solely assigned as Acidovorax by LCA (A) or BH (B) of different regions. The tags were extracted and then checked with RDP Classifier at confidence thresholds of 80%. The RDP results of Comamonadaceae at family level and Burkholderiales at order level were cataloged as accurate assignments. The V12 amplicons had no tag that was solely assigned by BH. (DOCX) [file pone.0076185.s001.docx]

**Supporting information for**

**Taxonomic precision of different hypervariable regions of 16S rRNA gene and annotation methods for functional bacterial groups in biological wastewater treatment**

Feng Guo, Feng Ju, Lin Cai and Tong Zhang*

Environmental Biotechnology Laboratory, The University of Hong Kong, Hong Kong SAR, China

Address: Environmental Biotechnology Lab, Department of Civil Engineering, The University of Hong Kong, Pokfulam Road, Hong Kong SAR, China

*Correspondence author

Email: zhangt@hku.hk

Tel: +852-28578551

Fax: +852-25595337

**Figures**

**Figure S1**

**Tables**

**Table S1 List of Primers sets used in trimming the full length 16S rDNA sequences into variable regions and PCR**

| Variable regions | Forward primer | Reverse primer | Coverage in RDP Probe Match^*^ | Average length^c^  (bp) |
| --- | --- | --- | --- | --- |
| V12 | AGAGTTTGATCCTGGCTCAG | TGCTGCCTCCCGTAGGAGT | 93.2%/97.2% | 311 |
| V34 | ACTCCTACGGGAGGCAGCAG | TACNVGGGTATCTAATCC | 84.9%/96.4% | 419 |
| V56 | ATTAGATACCCNGGTAG | CGACAGCCATGCANCACCT | 35.5%/96.6% | 248 |
| V789 | GYAACGAGCGCAACCC | GNTACCTTGTTACGACTT | 86.1%/96.4% | 370 |

^*^ The coverages of primer sets were determined in RDP ‘Probe Match’ module. The first one is percentage of perfect matched references and the second one is under allowing one mismatch. For V12 and V789, only one primer (i.e. reverse primer of V12 and forward primer of V789) was tested for its single coverage, respectively. Only reference sequences with high quality and over 1,200 bp belonging to bacteria domain were involved in this analysis.

^c^ Average lengths of trimmed segments (only for the selected functional genera) were given by the Pyro Pipeline of RDP.

**Table S2 Divergence between best-hit and the lowest common ancestor methods for moderately abundant genera**

| Genus | Abundance^a^  In % | Index A^b^  % | | | | | | | | Index B^c^ % | | | | | | | |
| --- | --- | --- | --- | --- | --- | --- | --- | --- | --- | --- | --- | --- | --- | --- | --- | --- | --- |
|  |  | V12 | V21 | V34 | V43 | V56 | V65 | V789 | V987 | V12 | V21 | V34 | V43 | V56 | V65 | V789 | V987 |
| *Zoogloea* | 4.28 | 99.3 | 98.7 | 98.6 | 95.6 | 99.8 | 99.5 | 51.2 | 78.2 | 92.4 | 89.4 | 73.3 | 84.8 | 92.3 | 84.8 | 83.0 | 84.6 |
| *Dechloromonas* | 3.41 | 98.5 | 98.1 | 90.0 | 85.1 | 85.9 | 89.6 | 80.9 | 77.6 | 76.7 | 74.4 | 70.8 | 80.3 | 82.0 | 76.3 | 57.5 | 55.2 |
| *Nitrospira* | 1.68 | 100.0 | 100.0 | 100.0 | 100.0 | 100.0 | 100.0 | 100.0 | 100.0 | 97.6 | 97.3 | 85.3 | 93.3 | 100.0 | 98.6 | 80.8 | 86.0 |
| *Trichococcus* | 1.62 | 100.0 | 100.0 | 100.0 | 100.0 | 100.0 | 100.0 | 100.0 | 100.0 | 98.8 | 99.1 | 89.3 | 97.2 | 99.8 | 98.3 | 89.1 | 83.8 |
| *Clostridium* | 1.44 | 93.7 | 98.6 | 99.5 | 100.0 | 90.9 | 92.4 | 95.7 | 86.9 | **30.0** | **32.9** | 85.7 | 92.1 | 75.0 | 86.4 | 58.8 | 68.9 |
| *Tetrasphaera* | 1.04 | 99.2 | 99.6 | **46.5** | **0.0** | **1.4** | **3.0** | **0.0** | **0.5** | **43.1** | **43.8** | 93.9 | **0.0** | **30.8** | **50.0** | **0.0** | **8.3** |
| *Acinetobacter* | 0.96 | 96.5 | 98.0 | 100.0 | 100.0 | 99.4 | 100.0 | 99.5 | 96.5 | 90.5 | 93.5 | 90.8 | 96.5 | 96.5 | 97.3 | 82.6 | 81.5 |
| *Acidovorax* | 0.94 | 99.3 | 98.3 | 95.3 | 90.8 | **27.6** | **14.5** | **9.5** | **9.2** | 94.6 | 93.6 | 77.2 | 87.9 | 97.6 | 94.7 | **45.3** | **31.3** |
| *Haliscomenobacter* | 0.83 | 100.0 | 100.0 | 100.0 | 100.0 | 100.0 | 100.0 | 100.0 | 100.0 | 62.1 | 61.9 | 91.1 | 93.8 | 100.0 | 98.5 | 83.3 | 76.7 |
| *Rhodobacter* | 0.81 | **48.4** | **48.9** | 92.7 | 60.9 | **4.7** | **47.0** | **20.5** | **20.6** | **19.0** | **21.5** | **42.5** | 69.2 | **8.1** | **26.7** | **34.0** | **28.0** |
| *Arcobacter* | 0.71 | 100.0 | 100.0 | 100.0 | 99.6 | 100.0 | 100.0 | 100.0 | 100.0 | 97.4 | 97.6 | 93.2 | 95.8 | 100.0 | 100.0 | 93.8 | 85.0 |
| *Mycobacterium* | 0.70 | 99.4 | 98.2 | 96.8 | 94.1 | 100.0 | 97.1 | 55.7 | 88.3 | 60.3 | 51.4 | **48.1** | 57.1 | 83.0 | 82.4 | 71.1 | 86.8 |
| *Thauera* | 0.70 | 98.8 | 99.7 | 100.0 | 100.0 | 100.0 | 99.7 | 95.9 | 92.7 | 91.5 | 93.3 | 82.8 | 91.2 | 99.1 | 98.3 | 89.4 | 82.9 |
| *Nitrosomonas* | 0.55 | 100.0 | 100.0 | 100.0 | 91.2 | 100.0 | 97.7 | **6.4** | **0.0** | 71.9 | 70.6 | 57.0 | 71.3 | 75.5 | 72.5 | **21.4** | **0.0** |
| *Curvibacter* | 0.47 | **0.0** | **0.0** | **0.0** | **0.0** | **0.0** | **0.0** | **2.0** | **1.3** | **0.0** | **0.0** | N.D. | **0.0** | N.D. | N.D. | **33.3** | 66.7 |
| *Caldilinea* | 0.32 | 100.0 | 100.0 | 100.0 | 100.0 | 100.0 | 100.0 | 100.0 | 100.0 | 88.9 | **50.0** | 52.5 | 59.5 | **42.3** | 60.7 | **42.0** | 52.8 |
| *Azoarcus* | 0.31 | N.D.^d^ | N.D. | **0.0** | **0.0** | N.D. | N.D. | **5.6** | **4.2** | **0.0** | N.D. | **0.0** | **0.0** | **0.0** | **0.0** | **33.3** | **33.3** |
| *Leptospira* | 0.28 | 100.0 | 100.0 | 100.0 | 100.0 | 100.0 | 100.0 | 100.0 | 100.0 | 59.0 | 66.2 | 81.5 | 86.3 | 94.1 | 87.5 | 87.7 | 73.1 |
| *Aeromonas* | 0.28 | 98.9 | 98.7 | 100.0 | 100.0 | 54.8 | 63.3 | 100.0 | 100.0 | 90.7 | 87.4 | 81.1 | 83.6 | 75.4 | 92.7 | 70.4 | 68.1 |
| *Gordonia* | 0.27 | 88.6 | 88.0 | 98.3 | 100.0 | **13.0** | **12.2** | 87.7 | 93.8 | 89.9 | 91.0 | 89.1 | 88.0 | **46.2** | **45.2** | 85.1 | 97.8 |
| *Hyphomicrobium* | 0.25 | 100.0 | 100.0 | 98.4 | 79.2 | 90.4 | 89.7 | 77.8 | **40.6** | 73.3 | 68.8 | 60.6 | 63.2 | 81.0 | 85.4 | 68.3 | 61.9 |
| *Paracoccus* | 0.19 | **12.9** | **12.8** | 76.7 | 62.2 | 83.9 | 70.0 | 60.5 | 57.7 | **36.4** | **41.7** | 82.1 | 91.8 | 94.5 | 84.0 | 79.3 | 62.5 |
| *Pseudomonas* | 0.16 | 100.0 | 100.0 | 91.7 | 100.0 | 96.8 | 91.4 | 100.0 | 90.5 | 83.3 | 85.2 | 62.9 | **40.9** | 73.2 | 82.1 | **46.2** | **46.3** |
| *Comamonas* | 0.14 | 100.0 | 96.3 | 80.0 | **45.0** | **4.3** | **46.4** | 65.0 | 60.0 | 95.8 | 89.7 | 55.2 | 52.9 | **28.6** | **48.1** | **36.6** | **54.5** |
| *Streptococcus* | 0.13 | 100.0 | 100.0 | 100.0 | 100.0 | 100.0 | 100.0 | 96.2 | 100.0 | 100.0 | 100.0 | 79.5 | 92.9 | 96.3 | 96.9 | 81.0 | 84.5 |
|  |  |  |  |  |  |  |  |  |  |  |  |  |  |  |  |  |  |

^a^ Twenty-five genera accounted over 0.1% average abundance determined by all three methods were listed.

^b^Index A is the percentage of co-classified tags by BH and LCA to all BH-classified tags. This index is the indicator of potential overestimation by BH and underestimation by LCA. The lower value of index A suggests the more BH-classified tags are not recognized by LCA.

^c^Index B is the percentage of co-classified tags by BH and LCA to all LCA-classified tags. This index is the indicator of potential overestimation by LCA and underestimation by BH. The lower value of index B suggests the more LCA-classified tags are not recognized by BH. For both index, the percentages lower than 50% with boldface may be highly biased ones.

^d^No tags were found by BH (index A) or LCA (index B).
